# Long-term outcomes remain unchanged despite reduced glucocorticoid exposure in ANCA-associated vasculitis: the multicentre REVEAL cohort study

**DOI:** 10.3389/fimmu.2026.1807423

**Published:** 2026-04-02

**Authors:** Mayu Shiomi, Ryu Watanabe, Muneyuki Hatta, Shogo Matsuda, Takuya Kotani, Ayana Okazaki, Yuichi Masuda, Mikihito Shoji, Atsushi Manabe, Keiichiro Kadoba, Tsuneyasu Yoshida, Naoko Ito, Youhei Fujiki, Hirofumi Miyake, Wataru Yamamoto, Motomu Hashimoto

**Affiliations:** 1 Department of Clinical Immunology, Osaka Metropolitan University Graduate School of Medicine, Osaka, Japan; 2 Department of Internal Medicine (IV), Osaka Medical and Pharmaceutical University, Takatsuki, Japan; 3 Department of Rheumatology and Clinical Immunology, Graduate School of Medicine, Kyoto University, Kyoto, Japan; 4 Division of Clinical Immunology and Rheumatology, Yodogawa Christian Hospital, Osaka, Japan; 5 Department of General Internal Medicine, Tenri Hospital, Nara, Japan; 6 Department of Health Information Management, Kurashiki Sweet Hospital, Okayama, Japan

**Keywords:** ANCA-associated vasculitis, glucocorticoid tapering, glucocorticoid toxicity, prognosis, real-world evidence

## Abstract

**Background:**

The long-term prognosis of patients with anti-neutrophil cytoplasmic antibody–associated vasculitis (AAV) has historically been poor. In recent years, the widespread use of novel targeted therapies has led to an increased emphasis on reduced-dose glucocorticoid (GC) strategies; however, owing to regional differences in the frequency and clinical characteristics of AAV subtypes, contemporary real-world evidence on long-term outcomes in Japanese patient populations remains limited.

**Objectives:**

The primary outcome was to elucidate subtype-specific clinical characteristics and five-year overall and relapse-free survival in anti-neutrophil cytoplasmic antibody (ANCA)-associated vasculitis. Secondary outcomes included temporal changes in treatment practices and prognosis, stratified by year of diagnosis.

**Methods:**

We conducted a multicentre retrospective study using data from the REVEAL cohort. A total of 460 newly diagnosed, treatment-naïve cases were included (microscopic polyangiitis (MPA), n = 283; granulomatosis with polyangiitis (GPA), n = 66; eosinophilic granulomatosis with polyangiitis (EGPA), n = 111). Clinical characteristics, five-year relapse-free and overall survival were evaluated. To assess temporal changes in treatment practices and prognosis, patients were stratified by median year of diagnosis (2018) into pre-2018 and post-2019 groups.

**Results:**

MPA showed the poorest prognosis among AAV subtypes, with a five-year overall survival rate of 67.6%. Older age and impaired renal function were independently associated with increased mortality (*P* < 0.001 and *P* = 0.0079, respectively). Over time, GC exposure was significantly reduced in the post-2019 group (*P* = 0.012), accompanied by fewer infection-related hospitalisations (*P* < 0.001). However, five-year overall and relapse-free survival did not differ between the pre-2018 and post-2019 groups. Notably, BVAS at 6 and 12 months were significantly higher (*P* = 0.0070 and *P* = 0.0057, respectively), and vasculitis-related mortality was more frequent in the post-2019 group (*P* = 0.0057).

**Conclusion:**

MPA remained the AAV subtype with the poorest prognosis. Although GC exposure has decreased in recent years, this trend was not associated with improved clinical outcomes in the present cohort.

## 1 Introduction

Anti-neutrophil cytoplasmic antibody (ANCA)-associated vasculitis (AAV) is a necrotising vasculitis characterised by few or no immune complex deposition, affecting small vessels throughout the body and leading to multiorgan dysfunction. AAV comprises three major clinicopathological variants: microscopic polyangiitis (MPA), granulomatosis with polyangiitis (GPA), and eosinophilic granulomatosis with polyangiitis (EGPA) (1). These subtypes differ in their genetic backgrounds, ANCA specificity—either myeloperoxidase (MPO) or proteinase 3 (PR3)—pathogenic mechanisms, clinical phenotypes, and disease courses (2, 3). The long-term prognosis of patients with AAV has historically been poor compared with that of the general population, and treatment has conventionally relied on glucocorticoids (GC) and cyclophosphamide, irrespective of disease subtype. However, in addition to mortality directly attributable to vasculitis, adverse effects associated with these immunosuppressive therapies—particularly infections—have significantly impaired long-term outcomes in affected patients.

In recent years, therapeutic strategies for AAV have been increasingly refined and individualised with the broader adoption of novel molecular targeted agents, such as rituximab (RTX) and C5a inhibitors for MPA and GPA, and anti–interleukin-5 (IL-5) and interleukin-5 receptor (IL-5R) monoclonal antibodies for EGPA. In particular, given concerns about treatment-related toxicities associated with conventional regimens, the importance of reduced-dose GC strategies has been increasingly emphasised in contemporary practice (4, 5). Indeed, accumulating evidence from various regions of the world indicates that long-term outcomes for patients with AAV have gradually improved over time (6, 7).

However, the frequency and clinical characteristics of AAV subtypes vary across geographic regions (8), and contemporary real-world evidence regarding long-term outcomes in Japanese patient populations remains insufficient. Furthermore, given the increasing adoption of novel therapeutic agents such as C5a inhibitors and anti–IL-5/IL-5R antibody therapies, evaluating temporal changes in GC dose and their association with improvements in prognosis is crucial for informing future treatment strategies.

Therefore, in this study, we analysed subtype-specific clinical characteristics and long-term outcomes of AAV using real-world data from the multicentre REVEAL cohort study in Japan. In addition, we aimed to clarify temporal trends in GC dose and patient prognosis over time.

## 2 Materials and methods

### 2.1 Study design

This multicentre, retrospective, observational study was conducted using data from the REgistry of Vasculitis patients to establish rEAL-world evidence (REVEAL) cohort. The REVEAL cohort comprises 555 patients with MPA, GPA, or EGPA who were registered up to May 31, 2024, and received care at one of five participating centres: Osaka Metropolitan University, Kyoto University, Osaka Medical and Pharmaceutical University, Tenri Hospital, and Yodogawa Christian Hospital (9, 10).

### 2.2 Patients

Patients with MPA were classified according to the Chapel Hill Consensus definitions (1); those with GPA met the Watts’ algorithm (11) and the 2022 ACR/EULAR classification criteria (12); and those with EGPA fulfilled the Lanham criteria (13), the 1990 ACR classification criteria (14), and the 2022 ACR/EULAR classification criteria (15, 16). For patients with double-positive ANCA serology, the final diagnostic classification was made by the treating physician at each participating centre based on the overall clinical presentation and the relevant classification criteria. Patients whose clinical information at disease onset was unavailable, as well as those who were newly diagnosed but had already received GC or immunosuppressants, were excluded. Ultimately, 460 patients were included in the analysis, comprising 283 with MPA, 66 with GPA, and 111 with EGPA (**Supplementary Figure 1**). The distribution of ANCA serological status according to AAV phenotype is summarised in **Supplementary Table 1**. Histological confirmation by biopsy was obtained in some patients; the distribution of biopsy sites according to AAV phenotype is shown in **Supplementary Table 2**. All clinical information, laboratory data, and outcome measures were retrospectively extracted from electronic medical records.

This study was conducted in accordance with the Declaration of Helsinki (1964) and its subsequent amendments. The study protocol was approved by the ethics committees of all participating institutions, including Osaka Metropolitan University (approval no. 2023-027), Kyoto University (approval no. R1540), Osaka Medical and Pharmaceutical University (approval no. 1529), Tenri Hospital (approval no. C23-11), and Yodogawa Christian Hospital (approval no. 2023-070). Informed consent was obtained from all patients. However, because the data were anonymised, the requirement for informed consent was waived by the ethics committees of Kyoto University and Osaka Metropolitan University for patients whose medical visits to these institutions were recorded prior to 2021.

### 2.3 Data collection

The retrospectively extracted clinical data included demographic characteristics such as age at admission and sex; laboratory findings (white blood cell count, haemoglobin level, platelet count, albumin, creatinine, C-reactive protein (CRP), myeloperoxidase (MPO)-ANCA, and proteinase 3 (PR3)-ANCA); GC doses up to 24 months; and treatment history, including the use of immunosuppressants. Clinical manifestations were assessed using the 2009 Five-Factor Score (FFS) for prognostication (17), the Birmingham Vasculitis Activity Score (BVAS) version 3 for evaluating disease activity (18), and the Vasculitis Damage Index (VDI) for quantifying cumulative organ damage (19). Clinical outcomes included all-cause mortality, relapse, and infections requiring hospitalisation. Relapse was defined as recurrent, worsening, or new-onset disease activity that required treatment escalation, as determined by the treating physician. Relapses were further classified as major or minor according to a previous definition (20): major relapse involved potentially organ- or life-threatening disease, whereas minor relapse involved disease that was neither organ- nor life-threatening. The cause of death was determined using information recorded in the registry database and electronic medical records, and the classification was reviewed by the treating physician together with the first and corresponding authors. Deaths were categorised as vasculitis, infection, cardiovascular disease, malignancy, other, or unknown.

### 2.4 Outcomes

The primary outcome was to characterise clinical features stratified by AAV subtype and to evaluate five-year overall and relapse-free survival. The secondary outcomes were defined as changes in treatment practices and prognosis over time, stratified by year of diagnosis, and included GC dosing, the number of infection-related hospitalisations, VDI, and the five-year overall survival and relapse-free survival rates. GC dosing and tapering were determined by the treating physicians at each institution in accordance with routine clinical practice.

Patients included in the analysis were diagnosed between 2001 and 2024. To evaluate changes across diagnostic eras, they were stratified into two groups based on the median year of diagnosis (2018): those diagnosed between 2001 and 2018 (pre-2018 group) (n = 256) and those diagnosed between 2019 and 2024 (post-2019 group) (n = 204).

### 2.5 Statistical analysis

Data were presented as medians with interquartile ranges (IQRs) for continuous variables and as counts with percentages for categorical variables. Clinical characteristics were compared using the Kruskal–Wallis test for continuous variables and Pearson’s χ² test for categorical variables. Group comparisons of BVAS and VDI were performed using the Kruskal–Wallis test, and statistical significance was determined using the Bonferroni correction to adjust for multiple comparisons (21). Analyses of BVAS and VDI were performed using available cases at each timepoint, and the extent of missing data according to diagnostic era is summarised in **Supplementary Table 3**. Survival analyses were conducted using the Kaplan–Meier method, and differences between groups were assessed with the log-rank test. Longitudinal GC dose trajectories were evaluated using linear mixed-effects models, which appropriately account for repeated measurements and intra-individual correlations. Year-of-diagnosis group, timepoint, and their interaction were included as fixed effects, with each patient modelled as a random intercept. The cumulative GC dose over the first 24 months was estimated as the area under the dose–time curve using the trapezoidal rule. As a sensitivity analysis for informative missingness in GC dose data, inverse probability weighting (IPW) was applied at each timepoint, incorporating death, insufficient follow-up, and missing dose data after reaching the timepoint into the weighting model. Hazard ratios (HRs), 95% confidence intervals (CIs), and p-values for factors associated with mortality were calculated using Cox proportional hazards regression models. To evaluate cause-specific mortality, crude mortality rates were calculated as the number of deaths per 100 patient-years. Furthermore, to compare mortality across diagnostic periods, incidence rate ratios (IRRs) and their 95% CIs were calculated for each cause-of-death category. IRRs were estimated using exact Poisson tests, and p-values were obtained accordingly. In addition, competing-risk analyses were performed using Fine–Gray regression models. For major and minor relapse, death was treated as a competing event. For vasculitis-related death, non–vasculitis-related death and death of unknown cause were treated as competing events in the main analysis, whereas deaths of unknown cause were censored in the sensitivity analysis.

All statistical analyses and figure generation were performed using R software, version 4.4.2 (R Foundation for Statistical Computing, Vienna, Austria), and EZR, a graphical user interface for R (Saitama Medical Centre, Jichi Medical University, Saitama, Japan) (22). A two-sided p-value < 0.05 was considered statistically significant.

## 3 Results

### 3.1 Patient characteristics

Among the 555 patients with AAV enrolled in the REVEAL cohort, 283 with MPA, 66 with GPA, and 111 with EGPA were included in the analysis. The baseline clinical characteristics of the analysed patients are summarised in **Table 1**. At baseline, patients with MPA were the oldest and exhibited the lowest haemoglobin (Hb) and albumin (Alb) levels, whereas creatinine and CRP levels were the highest (all *P* < 0.001). Regarding ANCA subtypes, the frequency of MPO-ANCA positivity was highest in MPA (99.3%), while PR3-ANCA positivity was most frequent in GPA (45.5%) (both *P* < 0.001).

**Table 1:** Baseline clinical characteristics of MPA, GPA, and EGPA included in the analysis.

	MPA (n = 283)	GPA (n = 66)	EGPA (n = 111)	*P*-value
Age (years)	75.0 [69.0, 80.0]	69.0 [61.0, 76.5]	60.0 [51.0, 69.0]	<0.001
Female sex, n (%)	161 (56.9)	35 (53.0)	62 (55.9)	0.85
Disease duration (months)	44 [17, 82]	58 [18, 96]	53 [23, 94]	0.17
Five factor score 2009	2 [2, 2]	1 [1, 1]	1 [0, 1]	<0.001
Laboratory data				
White blood cell (/μL)	10630 [7900, 14038]	10300 [7420, 13300]	16200 [12730, 22300]	<0.001
Neutrophil (/μL)	8458 [5600, 11819]	7898 [4846, 11301]	6450 [4094, 8820]	<0.001
Lymphocyte (/μL)	1236 [861, 1714]	1137 [893, 1647]	1447 [994, 1930]	0.034
Hb (g/dL)	9.9 [8.7, 11.4]	11.8 [9.8, 12.7]	12.6 [11.5, 13.6]	<0.001
Alb (mg/dL)	2.6 [2.2, 3.2]	3.1 [2.4, 3.7]	3.0 [2.7, 3.5]	<0.001
Cr (mg/dL)	1.1 [0.7, 1.9]	0.7 [0.6, 0.9]	0.7 [0.6, 0.9]	<0.001
CRP (mg/dL)	8.3 [3.0, 12.8]	5.0 [1.3, 13.3]	3.3 [1.0, 6.5]	<0.001
MPO-ANCA, n (%)	281 (99.3)	25 (37.9)	45 (40.5)	<0.001
PR3-ANCA, n (%)	12 (4.2)	30 (45.5)	0 (0)	<0.001
BVAS total	14 [9, 19]	13 [8, 19]	17 [12, 22]	<0.001
General, n (%)	197 (69.6)	40 (60.6)	63 (56.8)	0.11
Cutaneous, n (%)	34 (12.0)	7 (10.6)	58 (52.3)	<0.001
Membrane/eye, n (%)	18 (6.4)	20 (30.3)	5 (4.5)	<0.001
ENT, n (%)	39 (13.8)	46 (69.7)	54 (48.7)	<0.001
Chest, n (%)	114 (40.3)	39 (59.1)	60 (54.1)	0.021
Cardiovascular, n (%)	7 (2.5)	1 (1.5)	19 (17.1)	<0.001
Abdominal, n (%)	2 (0.7)	2 (3.0)	7 (6.3)	0.022
Renal, n (%)	205 (72.4)	26 (39.4)	31 (28.0)	<0.001
Nervous system, n (%)	101 (35.7)	22 (33.3)	94 (84.7)	<0.001

Results are expressed as median [interquartile range] for continuous variables or the number (%) for nominal variables. Unless otherwise indicated, all data were collected at disease onset.

Alb, albumin; ANCA, anti-neutrophil cytoplasmic antibodies; BVAS, Birmingham Vasculitis Activity Score; Cr, Creatinine; CRP, C-reactive protein; ENT, Ear, Nose, and Throat; Hb, haemoglobin; MPO, myeloperoxidase; PR3, proteinase3.

Baseline BVAS was highest in EGPA (P < 0.001), and the proportions of patients presenting with cutaneous, cardiovascular, and neurological manifestations were also highest in this group (all *P* < 0.001). In contrast, renal involvement was more common in MPA, whereas mucosal and ear, nose, and throat (ENT) involvement was most frequent in GPA (all *P* < 0.001). Collectively, these findings indicate that distinct clinical features were observed among AAV subtypes at baseline.

### 3.2 Comparison of disease activity, organ damage, and prognosis among AAV subtypes

**Figure 1:**
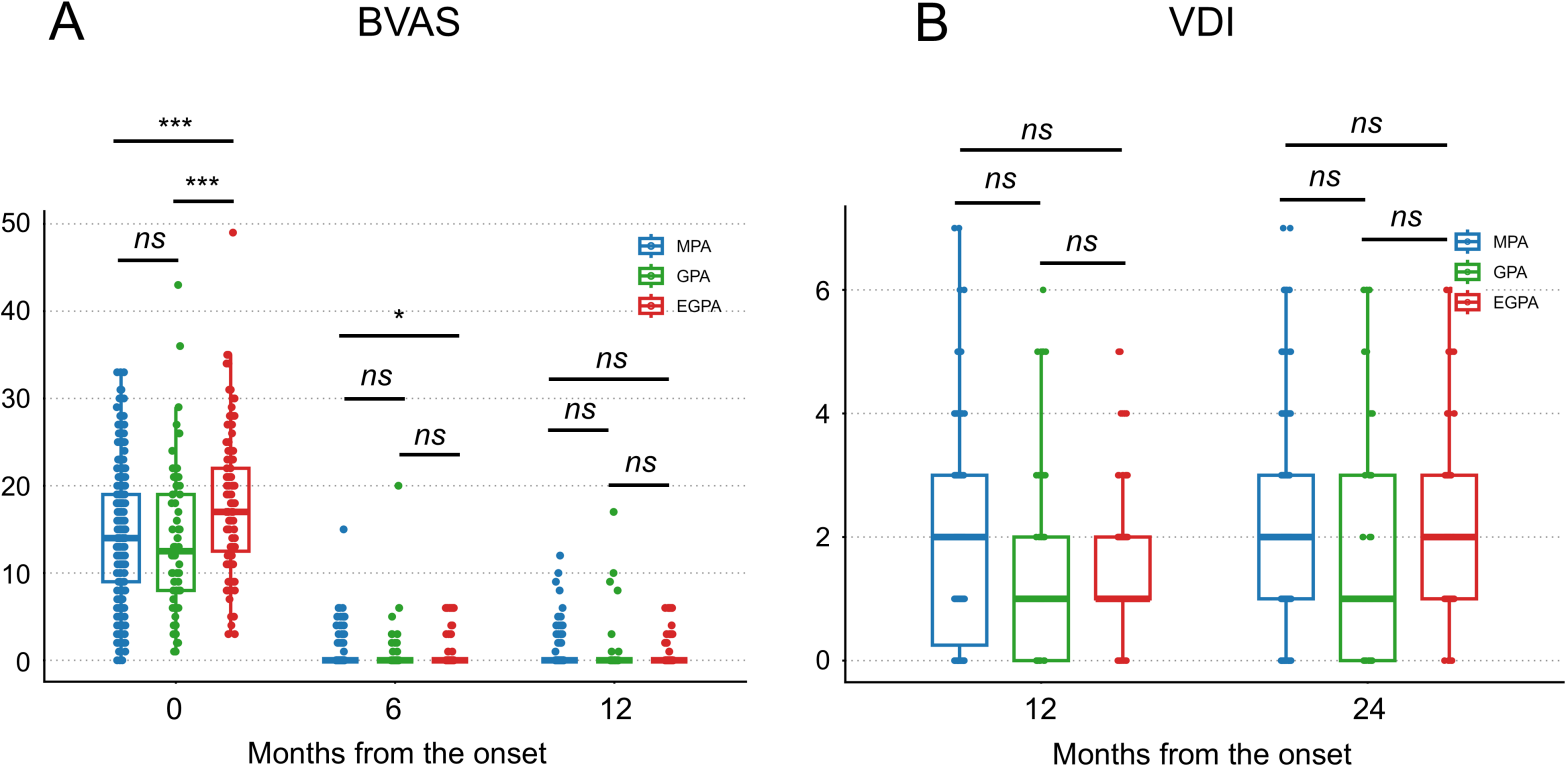
Longitudinal changes in BVAS and VDI among AAV subtypes. **(A)** EGPA showed higher BVAS at disease onset and at 6 months compared with other subtypes, whereas BVAS did not differ among groups at 12 months. **(B)** VDI was comparable among subtypes at both 12 and 24 months. ns, *P* ≥ 0.05; *, *P* < 0.05; ***, *P* < 0.001. AAV, anti-neutrophil cytoplasmic antibody–associated vasculitis; BVAS, Birmingham vasculitis activity score; EGPA, eosinophilic granulomatosis with polyangiitis; GPA, granulomatosis with polyangiitis; MPA, microscopic polyangiitis; VDI, Vasculitis Damage Index.

We next evaluated whether disease activity and organ damage differed over time among AAV subtypes (**Figure 1**). At disease onset, BVAS was highest in EGPA among the three subtypes (vs. MPA: *P* < 0.001; vs. GPA: *P* < 0.001), and EGPA remained significantly higher than MPA at 6 months (*P* = 0.042). However, no significant differences were observed among the three groups at 12 months (**Figure 1A**). In contrast, VDI did not differ significantly among the three subtypes at either 12 or 24 months (**Figure 1B**).

**Figure 2:**
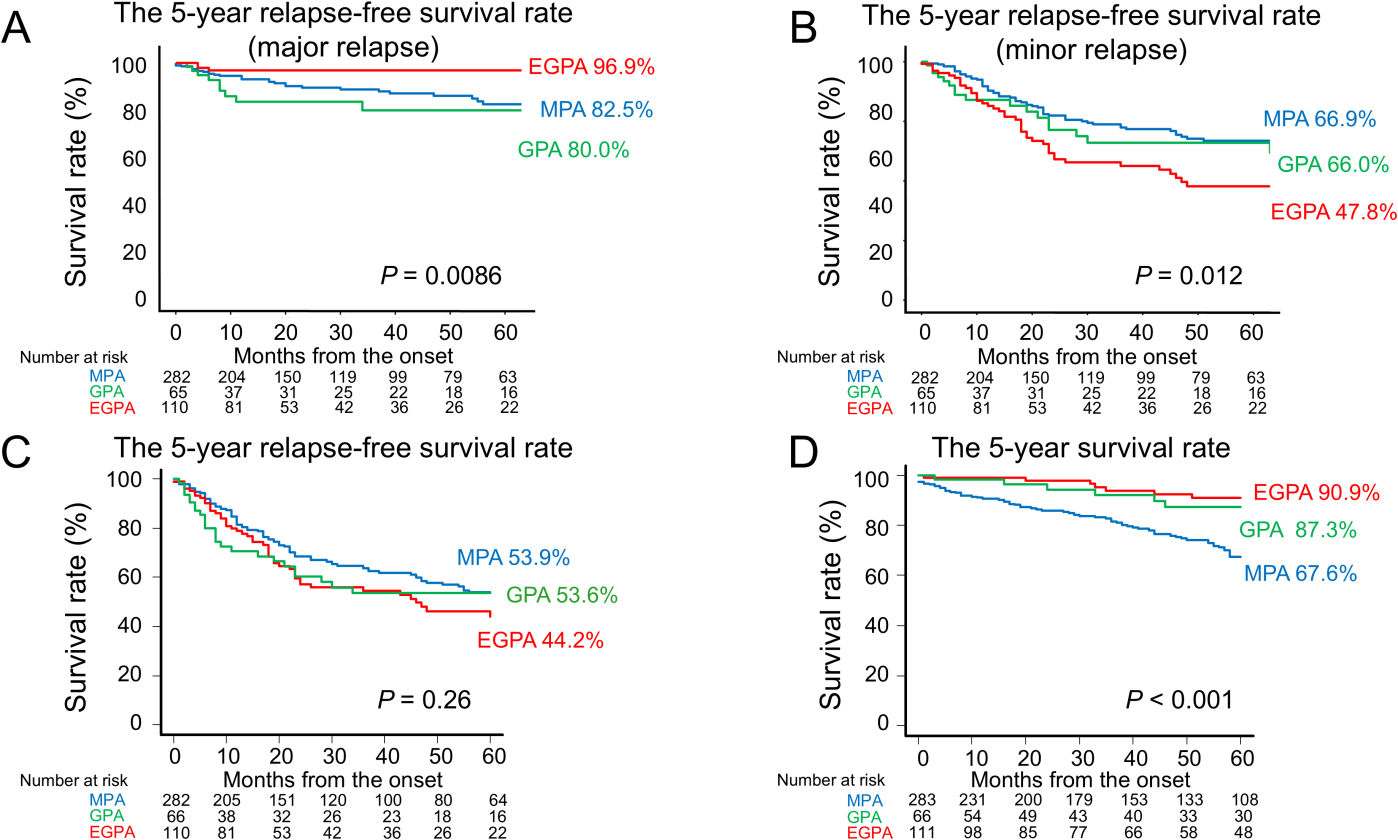
Five-year relapse-free survival and overall survival among AAV subtypes. **(A)** The 5-year relapse-free survival rate for major relapse was highest in EGPA (96.9%) and lowest in GPA (80.0%) (*P* = 0.0086). **(B)** The 5-year relapse-free survival rate for minor relapse was lowest in EGPA (47.8%) (*P* = 0.012). **(C)** No significant difference was observed in the 5-year relapse-free survival rate when all relapses were combined (*P* = 0.26). **(D)** The 5-year overall survival rate was lowest in MPA (67.6%) (*P* < 0.001). AAV, anti-neutrophil cytoplasmic antibody–associated vasculitis; EGPA, eosinophilic granulomatosis with polyangiitis; GPA, granulomatosis with polyangiitis; MPA, microscopic polyangiitis.

Regarding long-term outcomes, the five-year relapse-free survival rate for major relapse was significantly lower in GPA (80.0%) and higher in EGPA (96.9%) (*P* = 0.0086) (**Figure 2A**). For minor relapse, the five-year relapse-free survival rate was significantly lower in EGPA (47.8%) (*P* = 0.012) (**Figure 2B**). When all relapses were combined, the five-year relapse-free survival rates were 53.9% for MPA, 53.6% for GPA, and 44.2% for EGPA, with no significant difference among the groups (*P* = 0.26) (**Figure 2C**). In contrast, the five-year overall survival rates were 67.6% for MPA, 87.3% for GPA, and 90.9% for EGPA, indicating a significantly higher mortality rate in MPA (*P* < 0.001) (**Figure 2D**).

### 3.3 Multivariable Cox regression analysis of factors associated with mortality

Given the significantly lower survival rate observed in MPA, we performed multivariable Cox regression analyses to identify factors associated with mortality (**Table 2**). Ten variables were selected with reference to previous reports and considering the number of events. In the primary multivariable model, only older age and elevated serum creatinine remained independently associated with mortality (*P* < 0.001 and *P* = 0.0079, respectively). In an additional multivariable Cox model using GPA as the reference category,MPA was associated with higher all-cause mortality (adjusted HR 2.198, 95% CI 1.061–4.554; *P* = 0.034), whereas EGPA was not (adjusted HR 0.961, 95% CI 0.354–2.608; *P* = 0.94), after adjustment for age at onset, sex, baseline BVAS, serum creatinine, and alveolar haemorrhage. In a serotype-based sensitivity analysis restricted to patients with MPA or GPA, no significant difference in all-cause mortality was observed between MPO-only and PR3-only serotypes in either the unadjusted model or the model adjusted for baseline disease severity factors (**Supplementary Table 4**).

**Table 2:** Multivariable cox regression analysis of factors associated with mortality.

Variable	Hazard ratio	Lower 95%CI	Upper 95%CI	*P*-value
Age (years)	1.088	1.060	1.117	<0.001
Female (%)	0.763	0.507	1.147	0.19
Five factor score 2009	0.964	0.664	1.400	0.85
Hb (g/dL)	1.013	0.900	1.141	0.83
Alb (mg/dL)	0.970	0.664	1.417	0.87
Cr (mg/dL)	1.156	1.039	1.286	0.0079
MPO-ANCA positive (vs negative)	0.895	0.488	1.641	0.72
Alveolar hemorrhage (%)	1.304	0.684	2.488	0.42
BVAS at onset	1.018	0.988	1.049	0.25
ENT involvement (BVAS, %)	0.659	0.371	1.170	0.15
AAV subtype				
MPA vs GPA	2.198	1.061	4.554	0.034
EGPA vs GPA	0.961	0.354	2.608	0.94

In the analysis of AAV subtype, hazard ratios were adjusted for age at onset, sex, baseline BVAS, serum creatinine, and alveolar hemorrhage. GPA was used as the reference category.

Alb, albumin; ANCA, anti-neutrophil cytoplasmic antibodies; BVAS, Birmingham Vasculitis Activity Score; Cr, Creatinine; ENT, Ear, Nose, and Throat; Hb, haemoglobin; MPO, myeloperoxidase.

### 3.4 Changes in treatment practices and outcomes over time

**Figure 3:**
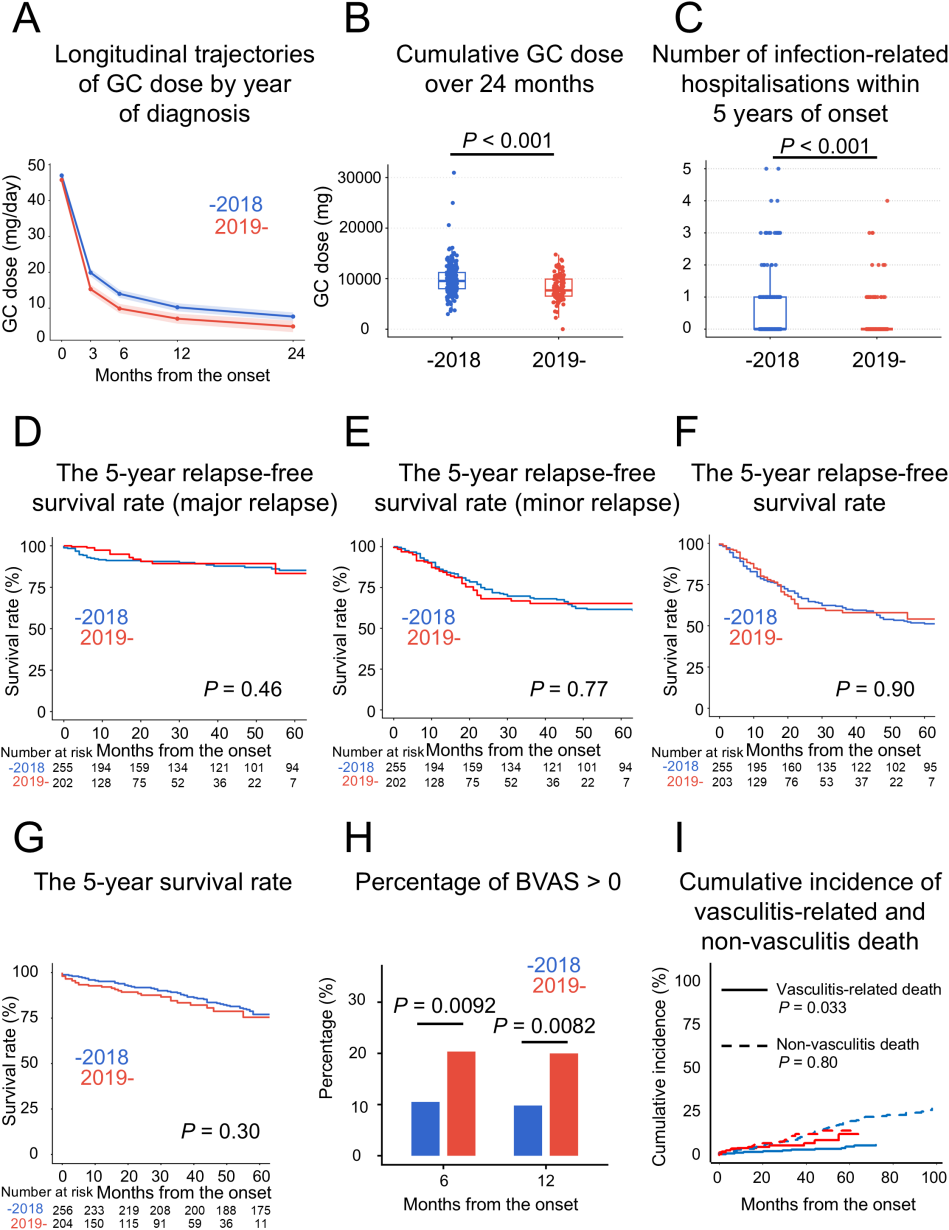
Glucocorticoid dose, infection-related hospitalisations, prognosis, disease activity, and vasculitis-related death stratified by year of diagnosis. Patients were stratified into the pre-2018 and post-2019 groups. **(A)** Longitudinal trajectories of GC dose by year of diagnosis. Estimated mean GC doses over time were compared using a linear mixed-effects model. Patients diagnosed ≤2018 showed a significantly higher GC dose trajectory than those diagnosed ≥2019 (*P* for interaction = 0.012). **(B)** Cumulative GC dose over 24 months by year of diagnosis. Cumulative GC dose, calculated as the area under the dose–time curve using the trapezoidal rule, was significantly lower in patients diagnosed ≥2019 than in those diagnosed ≤2018 (median [IQR], 7, 672 [6, 548–9, 911] mg vs 9, 540 ([, 010–11, 216] mg; *P* < 0.001). **(C)** Number of infection-related hospitalisations within 5 years of disease onset, which was significantly lower in the post-2019 group (*P* < 0.001). **(D)** The 5-year relapse-free survival for major relapse, showing no significant difference between the two groups. **(E)** The 5-year relapse-free survival for minor relapse, showing no significant difference between the two groups. **(F)** The 5-year relapse-free survival for all relapses, showing no significant difference between the two groups. **(G)** The 5-year overall survival, showing no significant difference between the two groups. **(H)** Percentage of patients with BVAS > 0 at 6 and 12 months, which was significantly higher in the post-2019 group. **(I)** Cumulative incidence of vasculitis-related death according to year of diagnosis, with non–vasculitis-related death and death of unknown cause treated as competing events. The cumulative incidence of vasculitis-related death was significantly higher in the post-2019 group (*P* = 0.033), whereas no significant between-era difference was observed for non–vasculitis-related death (*P* = 0.80). BVAS, Birmingham Vasculitis Activity Score; GC, glucocorticoid.

We next examined the secondary outcomes, namely temporal changes in treatment practices and prognosis across diagnostic eras, by stratifying the cohort according to the median year of diagnosis (2018) into two groups: patients diagnosed between 2001 and 2018 (pre-2018 group) and those diagnosed between 2019 and 2024 (post-2019 group) (**Figure 3**). With respect to remission-induction therapy, the use of GC pulse therapy and intravenous cyclophosphamide (IVCY) did not differ between the groups; however, RTX was administered significantly more frequently in the post-2019 group (**Supplementary Table 5**). Notably, longitudinal analysis using linear mixed-effects models demonstrated a significant difference in GC dose trajectories between the pre-2018 and post-2019 groups (*P* for group × time interaction = 0.012) (**Figure 3A**). When stratified by AAV subtype, a significant group × time interaction was observed only in patients with MPA (*P* = 0.0012), whereas no significant interaction was detected in GPA (*P* = 0.18) or EGPA (*P* = 0.11) (**Supplementary Figure 2**; **Supplementary Table 6**), suggesting that the overall finding was primarily driven by patients with MPA. To assess the potential impact of informative missingness in GC dose data, we performed an IPW analysis in which death, insufficient follow-up, and missing dose data after reaching each timepoint were incorporated into the weighting model. Patterns of missing GC dose data by timepoint and diagnostic era are summarised in **Supplementary Table 7**. The results were consistent with those of the primary analysis, showing lower GC doses in the post-2019 group at 3, 6, 12, and 24 months. The corresponding IPW-estimated marginal mean doses and 95% confidence intervals are presented in **Supplementary Table 8**.

Consistent with the longitudinal findings, cumulative GC exposure over the first 24 months was significantly lower in the post-2019 group than in the pre-2018 group (median [IQR], 7, 672 [6, 548–9, 911] mg vs 9, 540 [8, 010–11, 216] mg; *P* < 0.001) (**Figure 3B**). In parallel, the number of infection-related hospitalisations within five years of onset was significantly lower in the post-2019 group (*P* < 0.001) (**Figure 3C**), and a similar trend was observed when the analysis was restricted to respiratory infections (*P* = 0.056) (**Supplementary Figure 3**).

In contrast, with regard to long-term outcomes, no significant between-era differences were observed in the five-year relapse-free survival rates for major relapse, minor relapse, or overall relapse (**Figures 3D–F**). Competing-risk analyses treating death as a competing event also showed no significant between-era differences in either major or minor relapse (**Supplementary Table 9**). Similarly, five-year overall survival did not differ significantly between the eras, either in the overall cohort or in analyses stratified by AAV subtype, including MPA, GPA, and EGPA (**Figure 3G**; **Supplementary Figure 4**). Unexpectedly, although the BVAS at onset did not differ between the groups, BVAS at 6 and 12 months were significantly higher in the post-2019 group (*P* = 0.0070 and *P* = 0.0057, respectively) (**Supplementary Figure 5**). In particular, the percentage of patients with BVAS > 0 was significantly higher in the post-2019 group at both 6 and 12 months (*P* = 0.0092 and *P* = 0.0082, respectively) (**Figure 3H**). VDI scores at 12 and 24 months were comparable between the groups (both *P* = 0.66) (**Supplementary Figure 6**).

Finally, to further examine the relationship between early GC exposure and subsequent outcomes, we performed patient-level exposure–response analyses using the area under the dose–time curve from 0 to 6 months as a measure of early GC exposure. After adjustment for age at onset, sex, baseline BVAS, serum creatinine, alveolar hemorrhage, and induction regimen, no significant associations were observed with BVAS > 0 at 6 months, BVAS > 0 at 12 months, or all-cause mortality (**Supplementary Table 10**).

### 3.5 Changes in causes of death by year of diagnosis

Although overall prognosis did not differ by year of diagnosis, we evaluated whether the distribution of causes of death had changed over time. Causes of death were categorised into six groups: vasculitis, infections, cardiovascular disease, malignancy, other, and unknown. During the observation period, 78 deaths occurred in the pre-2018 group (n = 256) and 29 deaths occurred in the post-2019 group (n = 204). When converted to crude mortality rates per 100 patient-years and compared between the two groups, no temporal differences were observed in mortality due to infections, cardiovascular disease, or malignancy. In contrast, mortality due to vasculitis was significantly higher in the post-2019 group (IRR = 3.240, *P* = 0.0057) (**Table 3**). The detailed classification of vasculitis-related deaths is summarised in **Supplementary Table 11**.

**Table 3:** Cause-specific mortality incidence rate ratios: 2001–2018 vs 2019–2024.

Cause of death	n		Crude mortality rate (/100 patient-years)		IRR (95% CI)	*P*-value
	≤2018 (n = 256)	≥2019 (n = 204)	≤2018 (n = 256)	≥2019 (n = 204)		
Vasculitis	14	12	0.81	2.62	3.240 (1.37, 7.54)	0.0057
Infections	35	12	2.02	2.62	1.295 (0.51, 2.56)	0.47
Cardiovascular	6	1	0.35	0.22	0.629 (0.013, 5.19)	1.0
Malignancy	8	3	0.46	0.65	1.416 (0.242, 5.96)	0.71
Other	4	1	0.23	0.22	0.944 (0.019, 9.55)	1.0
Unknown	11	0	0.64	0	0 (0, 1.05)	0.14
Total	78	29	4.51	6.33	1.40 (0.884, 2.18)	0.12

IRR, Incidence rate ratio.

To further account for potential bias related to deaths of unknown cause, we performed competing-risk analyses for vasculitis-related death. In the main analysis, non–vasculitis-related death and death of unknown cause were treated as competing events. In the sensitivity analysis, deaths of unknown cause were censored. The cumulative incidence curves from the main analysis are shown in **Figure 3I**. The cumulative incidence of vasculitis-related death was significantly higher in the post-2019 group (*P* = 0.033), whereas no between-era difference was observed for non-vasculitis-related death (*P* = 0.80). Fine–Gray competing-risk regression showed a similar trend toward a higher incidence of vasculitis-related death in the post-2019 group in both the main and sensitivity analyses. However, after adjustment for baseline disease severity and induction regimen, no statistically significant between-era difference was observed in the cumulative incidence of vasculitis-related death (main analysis: *P* = 0.097; sensitivity analysis: *P* = 0.099) (**Supplementary Table 12**).

## 4 Discussion

This study showed that, among the three AAV subtypes, mortality remained highest in MPA, with age and renal function identified as major prognostic factors. Furthermore, although GC doses have decreased in recent AAV treatment practice, this trend was not associated with improved clinical outcomes. Our findings also suggest that vasculitis activity may have been less adequately controlled in more recent years. Although no direct causal relationship can be established, these observations raise the possibility that an overly uniform GC tapering strategy may, in some patients, compromise adequate control of the underlying vasculitis.

The three subtypes of AAV are known to exhibit distinct and characteristic clinical features (3). In the present study, consistent with previous reports, renal involvement was most frequently observed in MPA, whereas mucosal and ENT involvement was predominant in GPA, and cardiac and neurological involvement was more common in EGPA. Notably, among all organ manifestations, neurological involvement in patients with EGPA showed the highest prevalence, occurring in 84.7%. The higher BVAS observed in EGPA at both baseline and 6 months may be explained by this high prevalence of neurological involvement.

In contrast, although no significant differences in the VDI were observed among the three subgroups, the five-year survival rate was markedly poorer in patients with MPA, at 67%, compared with the other subtypes. Since the 1990s, multiple studies have examined prognosis according to AAV subtype, consistently demonstrating that MPA is associated with the worst outcomes (17, 23–25). Moreover, several studies have sought to identify factors associated with mortality in AAV, frequently reporting advanced age and impaired renal function as key predictors of poor prognosis (23, 26–30). The identification of age and renal function as prognostic factors in the present study suggests that these variables have remained central determinants of outcome in AAV to date. By contrast, in the serotype-based sensitivity analysis, all-cause mortality did not differ significantly between the MPO-only and PR3-only serotypes. This may be partly explained by the relatively high proportion of MPO-only cases within the GPA group (36%), which may have attenuated differences between the serotype-defined groups.

Focusing on studies that have evaluated the prognosis of AAV over time, previous reports indicate that outcomes gradually improved up to approximately the 2010s (7, 25, 27, 31–33). Although AAV was historically regarded as a disease associated with high mortality, in recent years the disease course has been evolving from a fatal condition towards a chronic relapsing remitting disorder. This shift has largely been driven by the introduction and widespread adoption of targeted therapies, including RTX and C5a receptor inhibitors for MPA and GPA, as well as anti–IL-5/IL-5R monoclonal antibodies for EGPA (2).

In recent years, the importance of GC-related toxicity has been increasingly emphasised in the management of AAV. Previous studies using the glucocorticoid toxicity index (GTI) in patients with AAV have shown that higher cumulative GC exposure is associated with increased VDI scores and a higher incidence of infections (34–36). Against this background, therapeutic strategies aimed at minimising GC-related toxicity have been actively explored. In a randomised controlled trial comparing standard-dose and reduced-dose GC regimens in combination with RTX among patients with AAV without severe glomerulonephritis or alveolar haemorrhage, the reduced-dose regimen was non-inferior to the high-dose regimen with respect to remission induction at 6 months (37). Furthermore, in patients with MPA or GPA complicated by relatively severe active nephritis or alveolar haemorrhage, reduced-dose GC demonstrated efficacy comparable to that of standard-dose GC, while serious infections occurred significantly less frequently in the reduced-dose group (38). Consistently, GC-sparing strategies have been reported to reduce GC-related toxicity (39), and in EGPA, GC dose reduction has been incorporated as a key outcome measure, in addition to disease activity, in recent clinical trials (40, 41). In the present study, the use of conventional immunosuppressive agents, such as azathioprine and tacrolimus, showed a decreasing trend in the post-2019 group, whereas the use of RTX increased significantly (**Supplementary Table 5**).

Although GC tapering in patients with MPA and GPA has been reported to be slower in Japan than in other countries and standard tapering protocols (42, 43), our cohort demonstrated a significant temporal reduction in GC exposure, with lower GC doses observed in the post-2019 group compared with the pre-2018 group, particularly among patients with MPA. Furthermore, the number of infection-related hospitalisations was also significantly lower in the post-2019 group, suggesting that GC tapering strategies may have contributed to a reduction in infection risk.

However, with respect to prognosis, although no significant increase in relapse rates was observed in the post-2019 group compared with the pre-2018 group, this did not translate into an improvement in overall survival. In another previous analysis of the REVEAL cohort, all-cause mortality, relapse, and infections requiring hospitalisation did not differ significantly between patients who were able to taper GC doses to ≤10 mg/day at 6 months and those who were not (44). Notably, in the present study, BVAS at both 6 and 12 months were significantly higher in the post-2019 group than in the pre-2018 group, and the proportion of patients with BVAS > 0 was also significantly increased. Furthermore, cause-specific mortality analyses suggested a higher frequency of vasculitis-related deaths in more recently diagnosed patients, whereas no temporal difference was observed in mortality due to infections. Indeed, one report has indicated that a PEXIVAS-guided reduced-dose GC regimen was associated with a significantly higher incidence of adverse outcomes, including mortality, progression to end-stage kidney disease, and AAV progression prior to remission (45).

However, in the sensitivity analyses performed in the present study, early GC exposure was not significantly associated with subsequent vasculitis activity or mortality after adjustment for baseline disease severity and induction regimen (**Supplementary Table 10**). Moreover, although vasculitis-related death appeared to be more frequent in the more recent era, this difference did not remain statistically significant after adjustment for baseline disease severity and induction regimen (**Supplementary Table 12**). Taken together, these findings should be interpreted with considerable caution. Nevertheless, one possible hypothesis arising from the present study is that rapid GC tapering may not always be sufficient to maintain adequate vasculitis control.

This study has several limitations. First, patients were stratified into two groups according to the median year of diagnosis (pre-2018 and post-2019). Although this approach enabled a broad assessment of temporal trends in treatment practices and outcomes, residual confounding between the two groups cannot be fully excluded. In particular, factors that may have changed over time—such as treatment selection beyond GC use, diagnostic accuracy, referral patterns, and advances in supportive care—were not fully accounted for and may have influenced the observed outcomes. Therefore, the absence of improvement in long-term prognosis cannot be attributed solely to changes in GC exposure. Second, as this was a retrospective observational study, causal relationships between GC tapering strategies, disease control, and clinical outcomes cannot be directly established. In addition, because patients who died were no longer available for subsequent GC dose assessments, longitudinal analyses of GC exposure may have been affected by informative missingness, introducing potential survivor bias. Third, cumulative GC exposure over the first 24 months was estimated using the trapezoidal rule based on discrete time-point measurements. Accordingly, the calculated cumulative doses represent approximations rather than exact values and may not fully capture short-term dose fluctuations between visits. Fourth, the observed increase in vasculitis-related mortality among patients diagnosed after 2019 should be interpreted with caution. Because all participating institutions in the REVEAL cohort were tertiary referral centres, severe or refractory cases may have been overrepresented. In addition, the higher proportion of patients with an unknown cause of death in the pre-2018 group may have influenced the apparent temporal differences in cause-specific mortality. Therefore, caution is warranted when generalising these findings to the broader AAV patient population.

Nevertheless, a key strength of this study is the inclusion of patients diagnosed with AAV between 2001 and 2024, enabling a comprehensive analysis of recent outcomes across more than two decades of evolving diagnostic and therapeutic practices. By leveraging a multicentre retrospective cohort, this study provides valuable real-world evidence on temporal changes in treatment practices and outcomes in routine clinical care.

## 5 Conclusion

MPA remains the AAV subtype with the poorest prognosis. Although GC exposure has decreased in recent years, this change was not associated with improved clinical outcomes in the present cohort.

## Data Availability

The raw data supporting the conclusions of this article will be made available by the authors, without undue reservation.
