# Supplementary material for: Long-term outcomes remain unchanged despite reduced glucocorticoid exposure in ANCA-associated vasculitis: the multicentre REVEAL cohort study
Source: Front Immunol. 2026 Apr 2;17:1807423. doi: 10.3389/fimmu.2026.1807423 (PMC13083106; doi:10.3389/fimmu.2026.1807423)

Supplementary Material

# Supplementary Table

**Supplementary Table 1. Distribution of ANCA serological status according to AAV phenotype.**

| Phenotype | MPO-only | PR3-only | Double-positive | ANCA-negative |
| --- | --- | --- | --- | --- |
| MPA, n (%) | 270 (95.4) | 1 (0.4) | 11 (3.9) | 1 (0.4) |
| GPA, n (%) | 24 (36.4) | 28 (42.4) | 1 (1.5) | 9 (13.6) |
| EGPA, n (%) | 45(40.5) | 0 (0) | 0 (0) | 66 (59.5) |

AAV, anti-neutrophil cytoplasmic antibody–associated vasculitis; ANCA, anti-neutrophil cytoplasmic antibody; EGPA, eosinophilic granulomatosis with polyangiitis; GPA, granulomatosis with polyangiitis; MPA, microscopic polyangiitis; MPO, myeloperoxidase; PR3, proteinase 3.

**Supplementary Table 2. Biopsy sites with histological confirmation according to AAV phenotype.**

|  | MPA (n = 283) | GPA (n = 66) | EGPA (n = 111) |
| --- | --- | --- | --- |
| No biopsy | 162 (57.2%) | 20 (30.3%) | 34 (30.6%) |
| Renal | 76 (26.9%) | 8 (12.1%) | 7 (6.3%) |
| Lung | 13 (4.6%) | 20 (30.3%) | 4 (3.6%) |
| Skin | 1 (0.4%) | 6 (9.1%) | 54 (48.6%) |
| Nerve | 0 (0%) | 0 (0%) | 2 (1.8%) |
| Other | 23 (8.1%) | 13 (19.7%) | 14 (12.6%) |
| Unknown | 8 (2.8%) | 2 (3.0%) | 0 (0%) |

Data are presented as n (%). Some patients had histological confirmation from multiple biopsy sites; therefore, the sum of biopsy sites may exceed the total number of patients in each group. Percentages were calculated using the total number of patients in each AAV phenotype group as the denominator.

AAV, anti-neutrophil cytoplasmic antibody–associated vasculitis; EGPA, eosinophilic granulomatosis with polyangiitis; GPA, granulomatosis with polyangiitis; MPA, microscopic polyangiitis.

**Supplementary Table 3. Missing BVAS and VDI data according to era.**

|  | ≤2018 (n = 256) | ≥2019 (n = 204) | Overall (n = 460) |
| --- | --- | --- | --- |
| BVAS at onset | 1 (0.4%) | 0 (0%) | 1 (0.2%) |
| BVAS at 6 months | 28 (10.9%) | 37 (18.1%) | 65 (14.1%) |
| BVAS at 12 months | 32 (12.5%) | 59 (28.9%) | 91 (19.8%) |
| VDI at 12 months | 32 (12.5%) | 57 (27.9%) | 89 (19.3%) |
| VDI at 24 months | 45 (17.6%) | 95 (46.6%) | 140 (30.4%) |

BVAS, Birmingham Vasculitis Activity Score; VDI, Vasculitis Damage Index.

**Supplementary Table 4. Sensitivity analysis of all-cause mortality comparing MPO-only and PR3-only serotypes in patients with GPA or MPA.**

| Model | Hazard ratio | Lower 95%CI | Upper 95%CI | *P*-value |
| --- | --- | --- | --- | --- |
| Unadjusted | 1.076 | 0.520 | 2.226 | 0.84 |
| Adjusted | 0.607 | 0.283 | 1.299 | 0.20 |

Hazard ratios were estimated using Cox proportional hazards models, with PR3-only as the reference category. The adjusted model included age at onset, sex, baseline BVAS, serum creatinine, and alveolar hemorrhage. EGPA was excluded, and double-positive and ANCA-negative cases were not included in this analysis.

**Supplementary Table 5. Comparison of clinical characteristics and treatments between the pre-2018 and post-2019 groups.**

|  | ≤2018  (n = 256) | ≥2019  (n = 204) | *P*-value |
| --- | --- | --- | --- |
| Age (years) | 70.0 [63.0, 76.2] | 74.0 [65.0, 80.0] | <0.001 |
| Female sex, n (%) | 142 (55.5) | 116 (56.9) | 0.84 |
| Disease duration (months) | 82.0 [47.0, 111.0] | 24.0 [8.75, 44.0] | <0.001 |
| MPA, n (%) | 164 (64.1) | 119 (58.3) | 0.21 |
| GPA, n (%) | 37 (14.5) | 29 (14.2) | 1.0 |
| EGPA, n (%) | 55 (21.5) | 56 (27.5) | 0.15 |
| Five factor score 2009 | 2 [1, 2] | 2 [1, 2] | 0.16 |
| BVAS total at onset | 14.0 [9.5, 20.0] | 16.0 [11.0, 19.0] | 0.46 |
| BVAS total at 6 months | 0 [0, 0] | 0 [0, 0] | 0.0070 |
| BVAS total at 12 months | 0 [0, 0] | 0 [0, 0] | 0.0057 |
| VDI total at 12 months | 1 [1, 2] | 1 [1, 3] | 0.66 |
| VDI total at 24 months | 2 [1, 3] | 2 [1, 3] | 0.66 |
| GC pulse, n (%)^a^ | 79 (32.2) | 76 (38.0) | 0.23 |
| IVCY, n (%)^a^ | 88 (35.1) | 86 (42.2) | 0.15 |
| RTX, n (%)^a^ | 21 (8.2) | 35 (17.2) | 0.0040 |
| Azathioprine, n (%)^b^ | 139 (54.5) | 94 (46.1) | 0.089 |
| Methotrexate, n (%)^b^ | 11 (4.3) | 8 (3.9) | 1.0 |
| Mycophenolate mofetil, n (%)^b^ | 4 (1.6) | 5 (2.5) | 0.52 |
| Tacrolimus, n (%)^b^ | 14 (5.5) | 1 (0.5) | 0.0025 |
| Mizoribine, n (%)^b^ | 8 (3.1) | 1 (0.5) | 0.048 |
| cyclosporine, n (%)^b^ | 3 (1.2) | 0 (0) | 0.26 |

Results are expressed as median [interquartile range] for continuous variables or the number (%) for nominal variables. ^a^ At initial remission induction. ^b^ At remission maintenance.

BVAS, Birmingham Vasculitis Activity Score; EGPA, eosinophilic granulomatosis with polyangiitis; GC, glucocorticoid; GPA, granulomatosis with polyangiitis; IVCY, intravenous cyclophosphamide; MPA, microscopic polyangiitis; RTX, Rituximab; VDI, Vasculitis Damage Index.

**Supplementary Table 6**. **Fixed effects from linear mixed-effects model for longitudinal GC dose between the pre-2018 and post-2019 groups.**

| Effect | Numerator degrees of freedom | Denominator degrees of freedom | *F*-value | *P*-value |
| --- | --- | --- | --- | --- |
| **MPA+GPA+EGPA (n = 460)** |  |  |  |  |
| Group (≤2018 vs ≥2019) | 1 | 406.15 | 35.37 | <0.001 |
| Timepoint (0m, 3m, 6m, 12m, 24m) | 4 | 1520.77 | 1894.50 | <0.001 |
| Group × Timepoint | 4 | 1520.77 | 3.22 | 0.012 |
| **MPA (n = 283)** |  |  |  |  |
| Group (≤2018 vs ≥2019) | 1 | 247.27 | 22.66 | <0.001 |
| Timepoint (0m, 3m, 6m, 12m, 24m) | 4 | 926.20 | 1356.29 | <0.001 |
| Group × Timepoint | 4 | 926.20 | 4.57 | 0.0012 |
| **GPA (n = 66)** |  |  |  |  |
| Group (≤2018 vs ≥2019) | 1 | 63.21 | 11.32 | 0.0013 |
| Timepoint (0m, 3m, 6m, 12m, 24m) | 4 | 216.77 | 161.02 | <0.001 |
| Group × Timepoint | 4 | 216.77 | 1.58 | 0.18 |
| **EGPA (n = 111)** |  |  |  |  |
| Group (≤2018 vs ≥2019) | 1 | 92.90 | 4.02 | 0.048 |
| Timepoint (0m, 3m, 6m, 12m, 24m) | 4 | 372.56 | 488.52 | <0.001 |
| Group × Timepoint | 4 | 372.56 | 1.89 | 0.11 |

*F* and *P* values were derived from Type III tests using Satterthwaite’s approximation.

EGPA, eosinophilic granulomatosis with polyangiitis; GC, glucocorticoid; GPA, granulomatosis with polyangiitis; MPA, microscopic polyangiitis.

**Supplementary Table 7**. **Patterns of missing glucocorticoid dose data by timepoint and era.**

| Timepoint (months) | n | | Observed | | Missing | | | | | | | |
| --- | --- | --- | --- | --- | --- | --- | --- | --- | --- | --- | --- | --- |
|  |  |  |  |  | Total | | Death before timepoint | | Not reached while alive | | Missing while alive and reached | |
|  | ≤2018 | ≥2019 | ≤2018 | ≥2019 | ≤2018 | ≥2019 | ≤2018 | ≥2019 | ≤2018 | ≥2019 | ≤2018 | ≥2019 |
| 0 | 256 | 204 | 255 (99.6) | 204 (100.0) | 1 (0.4) | 0 (0) | 0 (0) | 0 (0) | 0 (0) | 0 (0) | 1 (0.4) | 0 (0) |
| 3 | 256 | 204 | 240 (93.8) | 181 (88.7) | 16 (6.2) | 23 (11.3) | 4 (1.6) | 7 (3.4) | 6 (2.3) | 12 (5.9) | 5 (2.0) | 4 (2.0) |
| 6 | 256 | 204 | 228 (89.1) | 168 (82.4) | 28 (10.9) | 36 (17.6) | 6 (2.3) | 13 (6.4) | 10 (3.9) | 26 (12.7) | 8 (3.1) | 2 (1.0) |
| 12 | 256 | 204 | 221 (86.3) | 145 (71.1) | 35 (13.7) | 59 (28.9) | 12 (4.7) | 14 (6.9) | 13 (5.1) | 43 (21.1) | 9 (3.5) | 3 (1.5) |
| 24 | 256 | 204 | 213 (83.2) | 105 (51.5) | 43 (16.8) | 99 (48.5) | 20 (7.8) | 20 (9.8) | 22 (8.6) | 81 (39.7) | 5 (2.0) | 3 (1.5) |

Values are presented as n (%), with percentages calculated using the era-specific denominator (n). “Death before timepoint” indicates death before the specified timepoint. “Not reached while alive” indicates that follow-up duration was shorter than the specified timepoint among patients who were alive. “Missing while alive and reached” indicates missing prednisolone dose data despite follow-up having reached the specified timepoint.

**Supplementary Table 8**. **Inverse probability weighting analysis of glucocorticoid dose according to era.**

| Timepoint (months) | n  (≤2018) | IPW mean GC (95% CI)  (≤2018) | n  (≥2019) | IPW mean GC (95% CI)  (≥2019) | IPW mean difference  (≥2019 − ≤2018) (95% CI) |
| --- | --- | --- | --- | --- | --- |
| 3 | 232 | 19.79 (19.02–20.52) | 179 | 15.39 (14.42–16.31) | −4.40 (−5.54–−3.16) |
| 6 | 221 | 13.93 (13.24–14.58) | 160 | 10.04 (9.30–10.74) | −3.89 (−4.98–−2.92) |
| 12 | 212 | 10.05 (9.22–11.14) | 142 | 7.26 (6.40–8.31) | −2.78 (−4.10–−1.27) |
| 24 | 200 | 7.16 (6.62–7.73) | 98 | 5.13 (4.45–5.84) | −2.03 (−2.87–−1.10) |

Glucocorticoid dose is expressed as the prednisolone-equivalent dose. Inverse probability weights were derived from timepoint-specific logistic regression models estimating the probability of observing glucocorticoid dose at each timepoint (R(t)=1) according to era, baseline severity covariates, induction therapy, and prior glucocorticoid dose. Missingness was stringently defined such that R(t)=0 included both failure to reach the timepoint (due to death or insufficient follow-up) and missing dose data despite follow-up having reached the timepoint. Ninety-five percent confidence intervals were estimated by patient-level bootstrap resampling. n indicates the number of observed glucocorticoid dose values at each timepoint.

IPW, inverse probability weighting.

**Supplementary Table 9**. **Fine–Gray competing-risk regression analysis of major and minor relapse according to era.**

| Outcome | Comparison | sHR (95% CI) | *P*-value |
| --- | --- | --- | --- |
| Major relapse | ≥2019 (ref: ≤2018) | 0.70 (0.36–1.35) | 0.28 |
| Minor relapse | ≥2019 (ref: ≤2018) | 1.03 (0.72–1.48) | 0.86 |

Subdistribution hazard ratios were estimated using Fine–Gray competing-risk regression models. For major relapse, death and minor relapse were treated as competing events; for minor relapse, death and major relapse were treated as competing events.

sHR, Subdistribution hazard ratio.

**Supplementary Table 10**. **Patient-level exposure–response analyses using early glucocorticoid exposure (AUC, 0–6 months).**

| Outcome | Measure | Estimate (95% CI) | *P*-value |
| --- | --- | --- | --- |
| BVAS > 0 at 6 months | Adjusted OR | 0.998 (0.990–1.01) | 0.64 |
| BVAS > 0 at 12 months | Adjusted OR | 0.993 (0.983–1.00) | 0.11 |
| All-cause mortality | Adjusted HR | 0.996 (0.989–1.00) | 0.24 |

Models were adjusted for age at onset, sex, baseline BVAS, serum creatinine, alveolar hemorrhage, and induction regimen (IVCY only, RTX only, both, or other). Early glucocorticoid exposure was quantified as the area under the curve from 0 to 6 months (AUC, 0–6 months), approximated using the trapezoidal rule based on prednisolone dose at baseline, 3 months, and 6 months.

AUC, area under the curve; BVAS, Birmingham Vasculitis Activity Score; HR, hazard ratio; IVCY, intravenous cyclophosphamide; OR, odds ratio; RTX, Rituximab.

**Supplementary Table 11**. **Classification of vasculitis-related death.**

|  | vasculitis-related deaths (n = 26) |
| --- | --- |
| Acute exacerbation of interstitial lung disease | 10 |
| Alveolar hemorrhage | 4 |
| Gastrointestinal hemorrhage | 3 |
| Intracerebral hemorrhage | 3 |
| Renal failure | 2 |
| Other (treating physician–judged disease worsening) | 4 |

**Supplementary Table 12**. **Fine–Gray competing-risk regression analysis of vasculitis-related death according to era.**

| Analysis | Model | sHR (95% CI) for ≥2019 vs ≤2018 | *P*-value |
| --- | --- | --- | --- |
| Main | Unadjusted | 2.22 (0.99–4.95) | 0.052 |
|  | Adjusted | 1.95 (0.89–4.27) | 0.097 |
| Sensitivity | Unadjusted | 2.21 (0.99–4.92) | 0.053 |
|  | Adjusted | 1.94 (0.88–4.24) | 0.099 |

Subdistribution hazard ratios were estimated using Fine–Gray competing-risk regression models, with vasculitis-related death as the event of interest. In the main analysis, non–vasculitis-related death and death of unknown cause were treated as competing events. In the sensitivity analysis, death of unknown cause was censored. Adjusted models included age at onset, sex, baseline BVAS, serum creatinine, alveolar hemorrhage, and induction therapy. Adjusted analyses were based on complete cases (n = 451).

sHR, Subdistribution hazard ratio.

# Supplementary Figures

**Supplementary Figure 1.** **Patient selection flowchart.** Among 555 patients with ANCA–associated vasculitis, patients were classified using the criteria shown in the flowchart (MPA: Chapel Hill Consensus; GPA: Watts’ algorithm and the 2022 ACR/EULAR classification criteria; EGPA: Lanham criteria, the 1990 ACR classification criteria, and the 2022 ACR/EULAR classification criteria). Patients were excluded if clinical information at disease onset was unavailable or if they had already received glucocorticoids or immunosuppressants at diagnosis. The final analytic cohort included 460 patients (MPA, n=283; GPA, n=66; EGPA, n=111).

ACR, American College of Rheumatology; ANCA, antineutrophil cytoplasmic antibody; EGPA, eosinophilic granulomatosis with polyangiitis; EULAR, European Alliance of Associations for Rheumatology; GPA, granulomatosis with polyangiitis; MPA, microscopic polyangiitis.

**
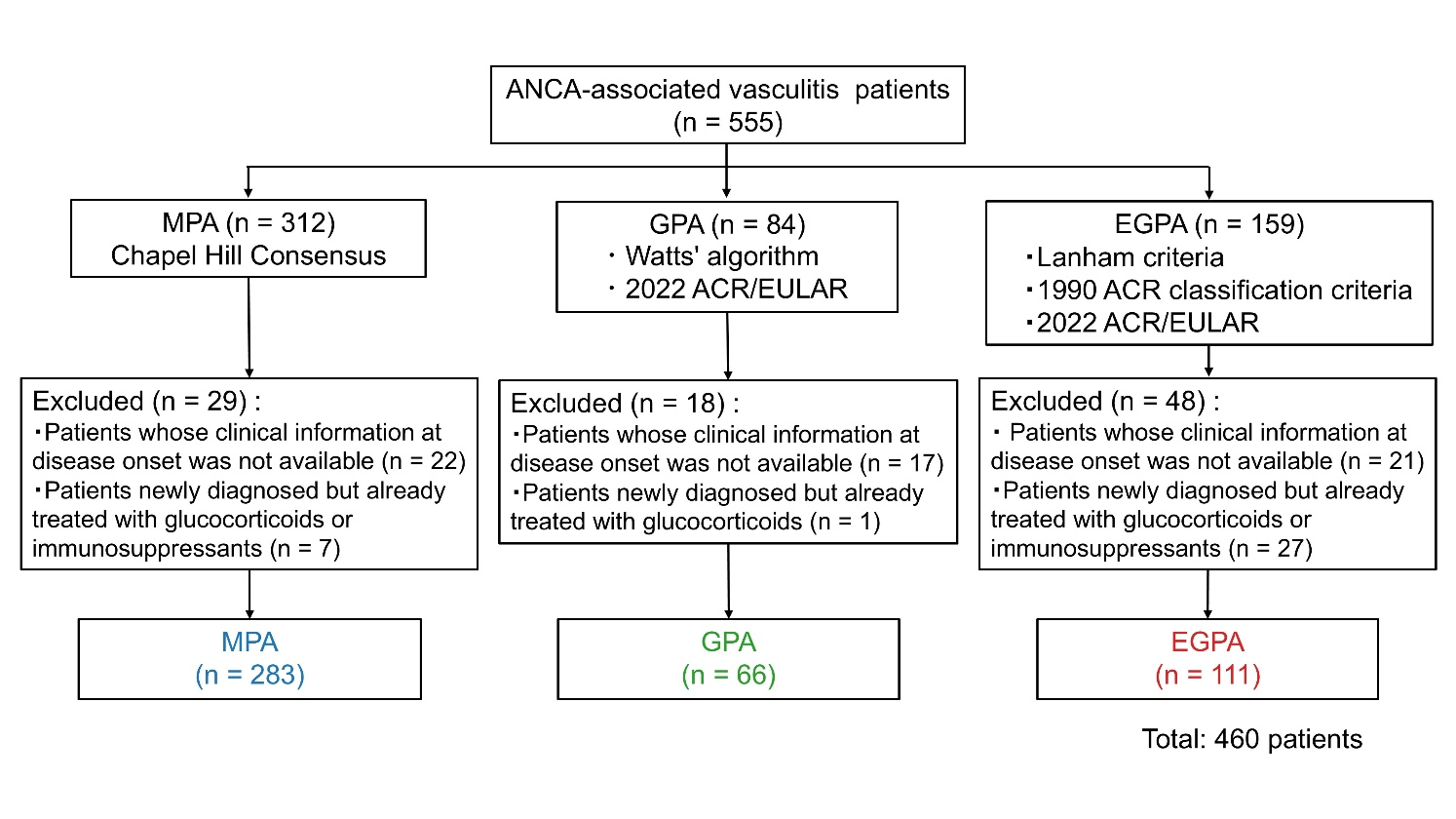
**

**Supplementary Figure 2. Longitudinal trajectories of GC dose by year of diagnosis in patients with MPA, GPA, and EGPA.** Estimated mean GC doses over time were compared using linear mixed-effects models. When analysed by subtype, a significant difference in GC dose trajectory between patients diagnosed ≤2018 and ≥2019 was observed only in MPA (A) (n = 283; *P* for interaction = 0.0012), whereas no significant group × time interaction was detected in GPA (B) (n = 66; *P* = 0.18) or EGPA (C) (n = 111; *P* = 0.11). Shaded areas indicate 95% confidence intervals.

EGPA, eosinophilic granulomatosis with polyangiitis; GC, glucocorticoid; GPA, granulomatosis with polyangiitis; MPA, microscopic polyangiitis.
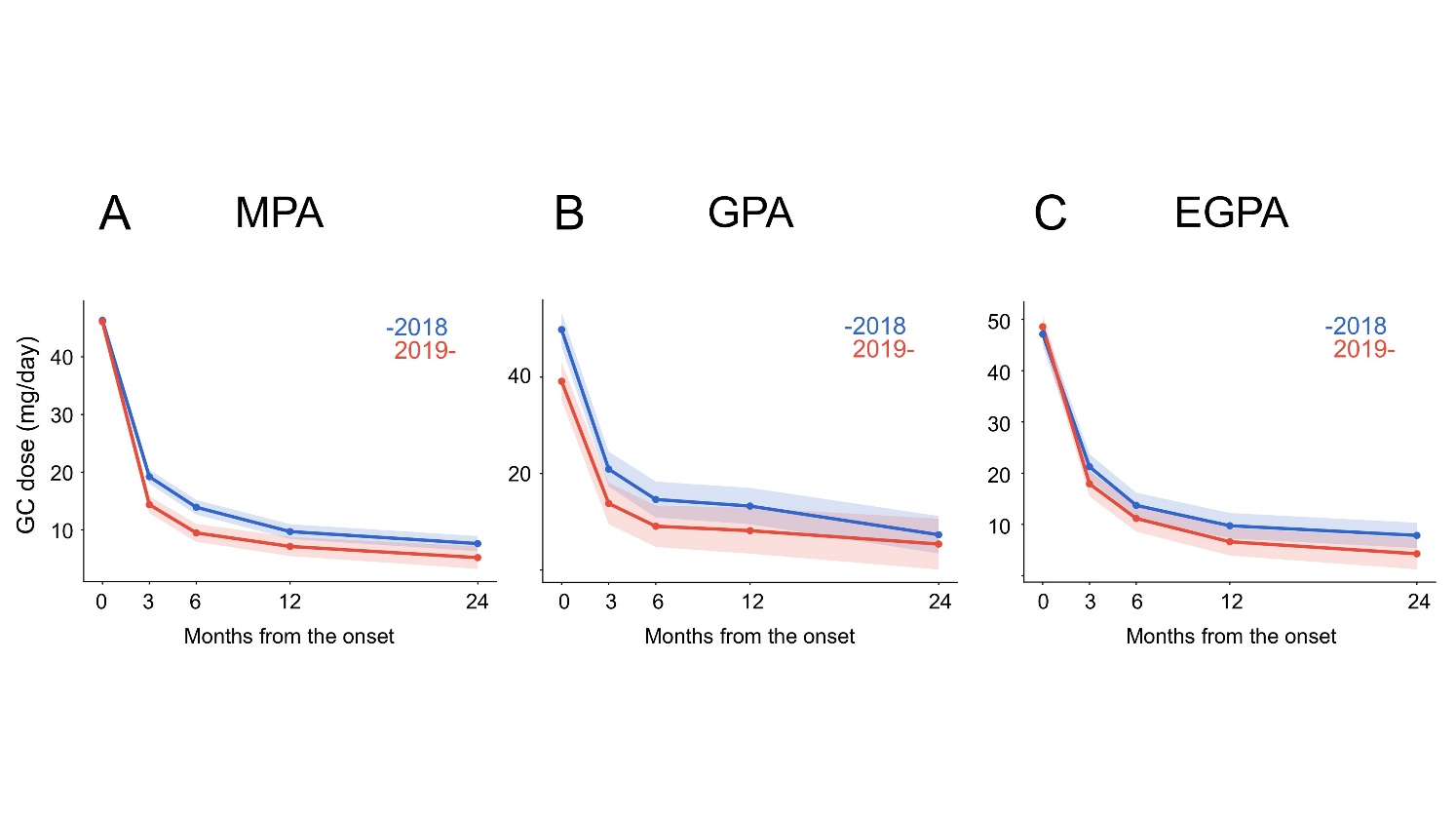


**Supplementary Figure 3. Number of respiratory infection–related hospitalisations within 5 years of onset stratified by year of diagnosis.** The number of respiratory infection–related hospitalisations within 5 years after disease onset showed a decreasing trend in the post-2019 group (*P* = 0.056).


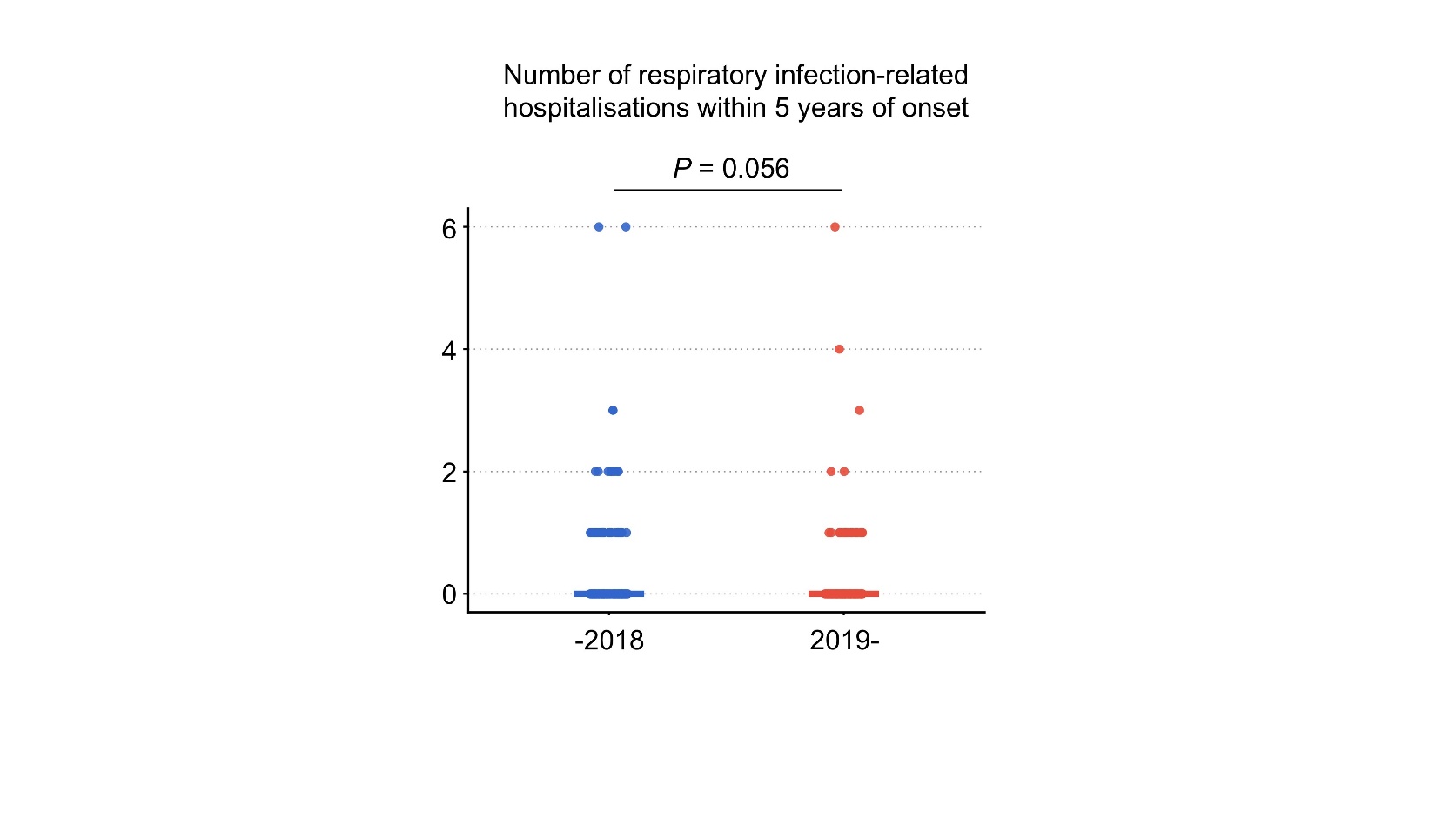


**Supplementary Figure 4. Five-year overall survival stratified by year of diagnosis in MPA, GPA, and EGPA.** Kaplan–Meier curves of overall survival over 60 months from disease onset are shown for (A) MPA, (B) GPA, and (C) EGPA. Patients diagnosed in 2018 or earlier are shown in blue, and those diagnosed in 2019 or later are shown in red. Five-year overall survival did not differ between the groups across all ANCA-associated vasculitis subtypes (MPA, *P* = 0.52; GPA, *P* = 0.068; EGPA, *P* = 0.57).

ANCA, antineutrophil cytoplasmic antibody; EGPA, eosinophilic granulomatosis with polyangiitis; GPA, granulomatosis with polyangiitis; MPA, microscopic polyangiitis.


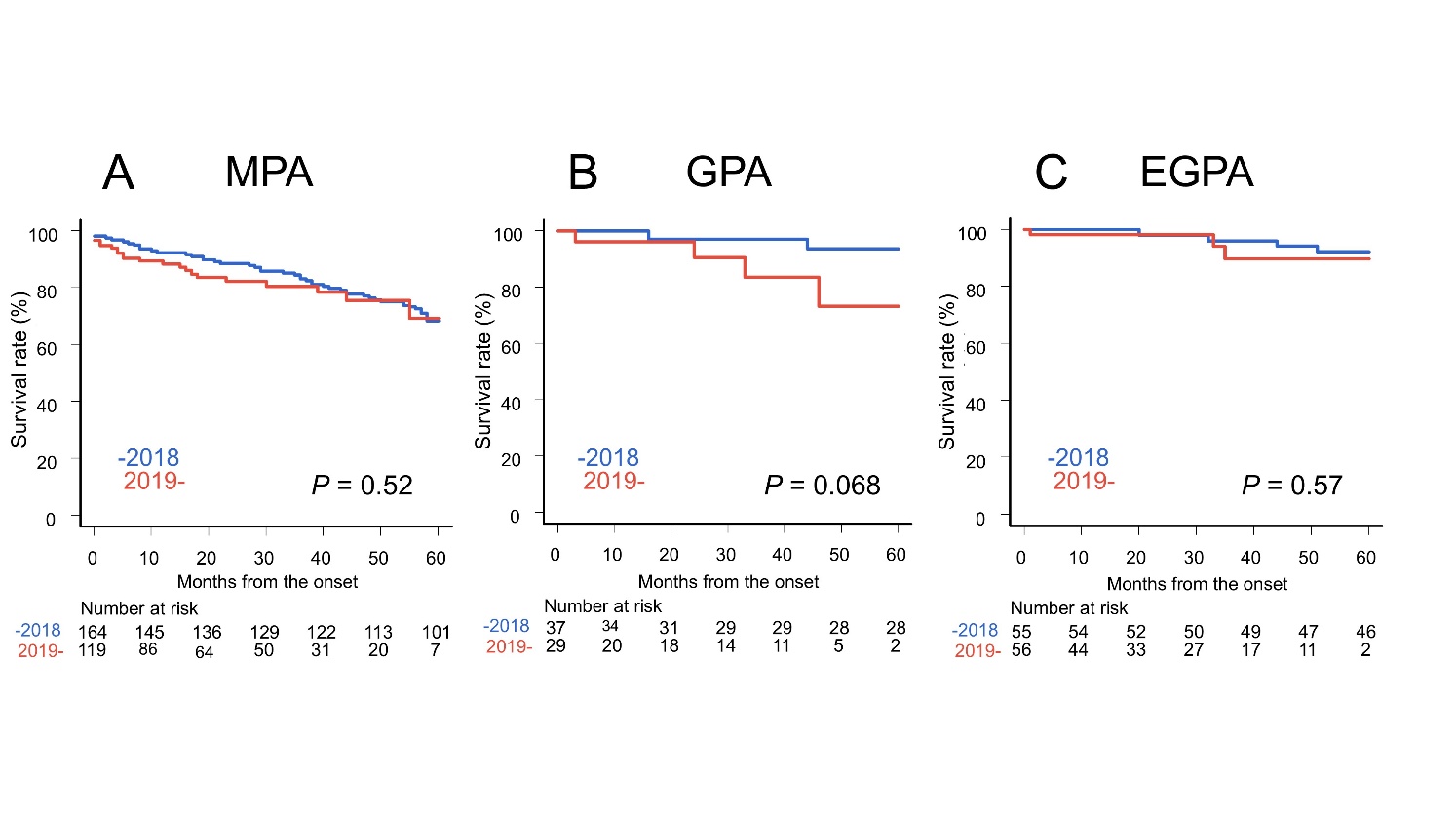


**Supplementary Figure 5. BVAS stratified by year of diagnosis.** BVAS did not differ between the pre-2018 and post-2019 groups at disease onset, whereas BVAS at 6 and 12 months was significantly higher in the post-2019 group (both *P* < 0.01).

BVAS, Birmingham vasculitis activity score. ns, *P* ≥ 0.05; *, *P* < 0.05; **, *P* < 0.01; ***, *P* < 0.001.


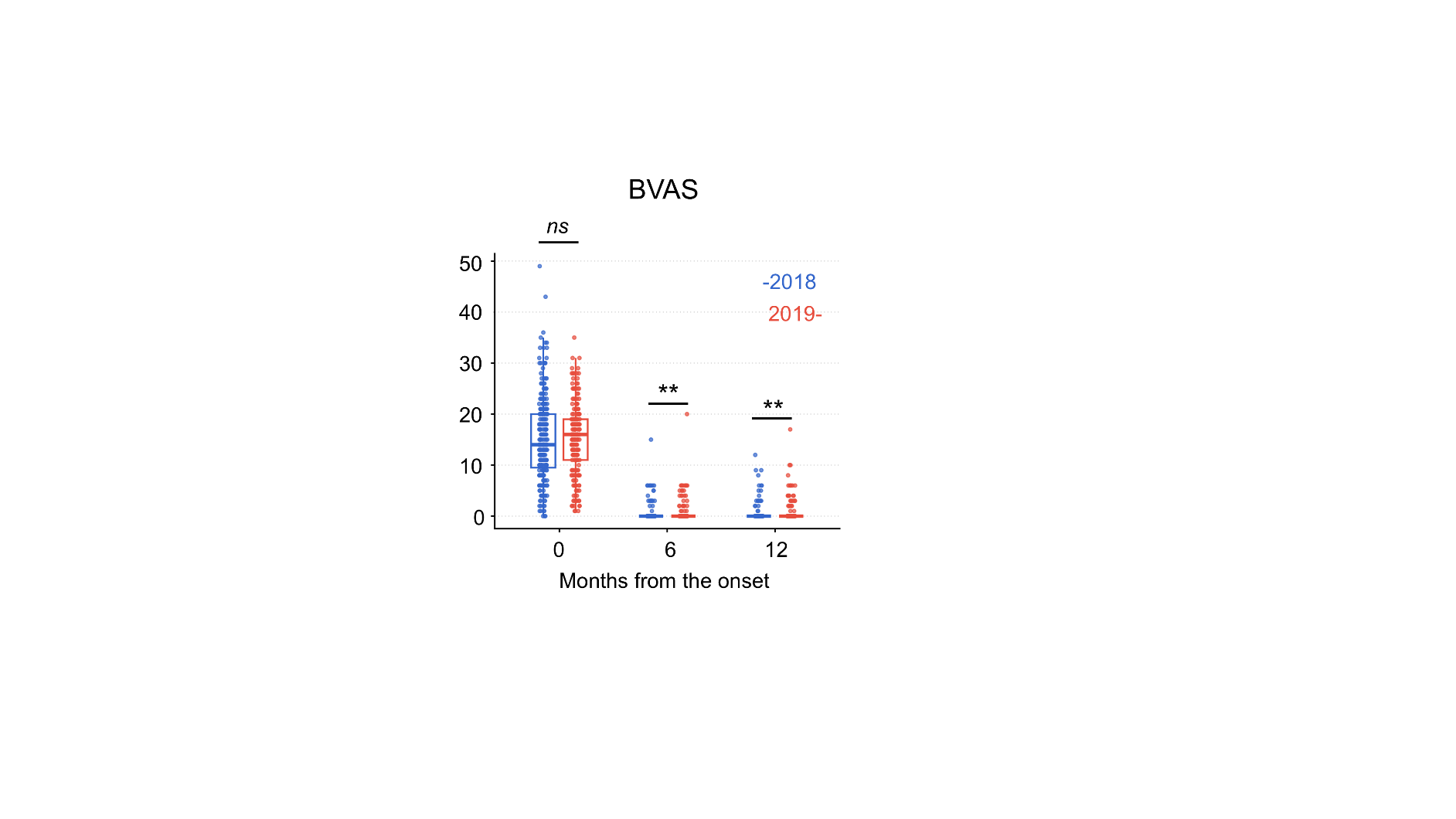


**Supplementary Figure 6. VDI stratified by year of diagnosis.** VDI scores at 12 and 24 months from disease onset were comparable between patients diagnosed in 2018 or earlier and those diagnosed in 2019 or later (both *P* = 0.66).

VDI, Vasculitis damage index. ns, *P* ≥ 0.05; *, *P* < 0.05; **, *P* < 0.01; ***, *P* < 0.001.


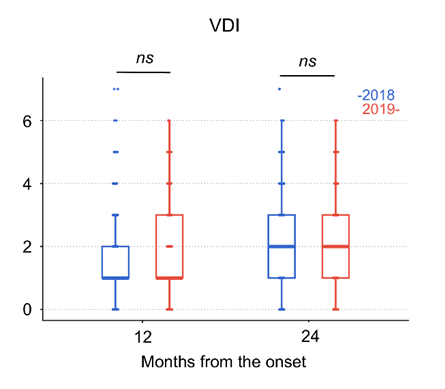

Supplement: Supplementary file 1 [file Table1.docx]
